# Affecting Cognition and Quality of Life via Aerobic Exercise in Alzheimer's Disease

**DOI:** 10.1177/0193945911420174

**Published:** 2011-09-12

**Authors:** Fang Yu, Nathaniel W. Nelson, Kay Savik, Jean F. Wyman, Maurice Dysken, Ulf G. Bronas

**Affiliations:** 1 University of Minnesota School of Nursing, Minneapolis; 2 University of St. Thomas, St. Paul, MN; 3 Minneapolis Veterans Affairs Medical Center, MN

**Keywords:** exercise, cognition, quality of life, dementia, aging

## Abstract

Aerobic exercise is a promising behavioral therapy for Alzheimer's disease (AD), yet few studies have investigated the effect of aerobic exercise on cognition in AD. The purpose of this pilot study was to examine the effect of 6-month aerobic exercise on the change in executive function, global cognition, quality of life (QOL), and depression in community-dwelling older adults with mild to moderate AD. A single group, repeated measures design with outcomes measured at baseline, 3 months, and 6 months was used. Results show that there were no significant changes in any measures except for depression (*p* = .026). There was a trend toward improvement in executive function and QOL with moderate effect sizes (ESs) and a trend toward deterioration in global cognition with moderate to large ESs. Randomized controlled trials are needed to evaluate the therapeutic effect of aerobic exercise in older adults with AD.

Alzheimer's disease (AD) is an epidemic health problem that affects more than 25 million people worldwide (Qiu, Kivipelto, & von Strauss, 2009) and 5.4 million Americans (Alzheimer's Association, 2011). Cognitive impairment is the cardinal feature of AD and results in limitations in activities of daily living, psychological and behavioral symptoms (American Psychiatric Association, 2000), poor quality of life (QOL), and escalating health care costs (Yu, Kolanowski, Strumpf, & Eslinger, 2006). Hence, developing interventions that could potentially ameliorate cognitive impairment is critical for altering AD trajectory and improving health care outcomes in this population. Aerobic exercise is one such potential intervention.

Recent studies in animals suggest that aerobic exercise might have a role in slowing cognitive decline in AD because it improves cerebral structure and function by improving neuronal survivability, regeneration, and function; upregulating growth factors; promoting vascularization; increasing hippocampal neurogenesis and volume; improving neuroplasticity; decreasing neuroinflammation; and decreasing AD neuropathologic β-amyloid load (Adlard, Perreau, Pop, & Cotman, 2004; Cotman & Berchtold, 2007). Meta-analyses of experimental studies showed that aerobic exercise improves cognition in adults without cognitive impairment. However, the effect sizes (ESs) vary: One meta-analysis showed moderate cognitive gains from aerobic exercise (Colcombe & Kramer, 2003), whereas others reported more modest cognitive gains (Angevaren, Aufdemkampe, Verhaar, Aleman, & Vanhees, 2008; Etnier et al., 1997; Etnier, Nowell, Landers, & Sibley, 2006; Smith et al., 2010). Although improvement was observed in all cognitive domains, executive function increased most strikingly (Colcombe & Kramer, 2003). Gain in global cognition is further observed in older adults with subjective memory loss (Lautenschlager et al., 2008) and in executive function, memory, and attention in those with mild cognitive impairment (Baker et al., 2010; Scherder et al., 2005; van Uffelen, Chinapaw, van Mechelen, & Hopman-Rock, 2008).

Nonetheless, there are few aerobic exercise studies in older adults with AD (Yu, 2011). They often had small sample sizes that limit the generalizability of the results. Few studies measured change in global cognition with mixed results, and no studies have ever measured executive function (Yu, 2011). Older adults with AD improved global cognition measured by the Mini-Mental State Examination (MMSE) from 16.3 at baseline to 19.8 posttraining (*p* < .001) following 5 to 12 weeks of aerobic exercise (*n* = 35; Rolland et al., 2000). A total of 3 months of cycling increased the scores on the Test of Attentional Matrix from 35.9 to 43, the Verbal Span Test from 2.9 to 3.8, the Supraverbal Span Test from 7.4 to 12.6, and the MMSE from 19.4 to 21.7 (all *p* < .001) from pre- to postexercise in 15 men with AD (Palleschi et al., 1996). A randomized controlled trial (*n* = 38) further showed that nursing home residents with AD improved global cognition that was measured by the French Rapid Evaluation of Cognitive Function from 26.8 to 30.4 (*p* < .01), whereas the control group dropped from 28.3 to 23.2 (*p* < .01; Kemoun et al., 2010). Although the 7 exercisers outperformed the 4 control individuals on global cognition, all 11 older adults performed equally and showed no change from pre- to post-exercise on other cognitive tests such as Wechsler Memory Scale–Revised Logical Memory I, Boston Naming Test, and Verbal Fluency Test (Arkin, 2001). No differences in the Boston Naming Test and Hopkins Verbal Learning Test scores were found between exercisers whose caregivers provided comprehensive exercises daily for 12 weeks at home and nonexercisers (*n* = 27; Steinberg, Leoutsakos, Podewils, & Lyketsos, 2009). The discrepancy in findings is largely attributable to prevailing methodological issues in existent studies, for example, low intensity and duration of aerobic exercise that may not be sufficient for generating cognitive benefits, inattention to differential sensitivity of various cognitive domains to aerobic exercise, and lack of comprehensive measures of cognition (Yu, 2011). The purpose of this pilot study was to examine the effect of 6-month aerobic exercise on the change in executive function, global cognition, QOL, and depression from baseline to month 3 and 6 in community-dwelling older adults with mild to moderate AD.

## Method

The pilot study used a single group, repeated measures design to test a 6-month, 3 times a week, individualized, moderate intensity cycling program in community-dwelling older adults with mild to moderate AD. Outcomes were collected on executive function, global cognition, QOL, and depression at baseline and again after participants completed 3 and 6 months of cycling, respectively. Participants received US$120 compensation (US$20 per month). The University of Minnesota's Institutional Review Board approved the study protocol.

### Sample

Four recruitment strategies were used to enroll participants from July 2008 to December 2009 as follows: (a) advertisements at senior newspapers, (b) referral by senior housing managers; (c) flyer distributions at senior housing and community events, and (d) seminars delivered by author F.Y. at AD support groups. Participants were qualified using the following inclusion criteria: have a probable AD diagnosis, live in the community such as home or residence where skilled nursing services are not provided, understand and speak English, age 60 years or above, and have medical clearance from the primary physician for aerobic exercise. Participants were excluded if their MMSE score was <12 during screening, had contraindications to exercise such as uncontrolled hypertension, manifested signs and symptoms that need to be evaluated by a physician, could not cycle, and had a recent history of unstable medical conditions.

### Setting

All study activities occurred at a retirement community in Saint Paul, Minnesota, where two Precor™ recumbent stationary cycles (Precor Inc., Woodinville, WA) were placed. Participants who lived outside the retirement community were transported to and from the retirement community by research staff.

### Data Collection

A trained undergraduate research assistant (RA) administered all measures for executive function, global cognition, QOL, and depression to participants at baseline and after participants completed 3 and 6 months of cycling. The RA also collected demographic data at baseline, including age, gender, race/ethnicity, marital status, education, and living arrangement.

Executive function was assessed with four measures: the Executive Interview (EXIT)-25 (Royall, Chiodo, & Polk, 2003), the Stroop Color-Word Test (Stroop, 1935), the Trail Making Test (TMT) Parts A and B (Mitrushina et al., 1994), and the Controlled Oral Word Association (COWA) Test (Ross, 2003). The EXIT-25 defines the behavioral sequelae of executive dysfunction, consists of 25 items, and can be administered in 15 min. Its score ranges from 0 to 50 with higher scores indicating greater executive dysfunction. The EXIT-25 has high internal consistency (Cronbach's α = .87) and interrater reliability (*r* = .90; Royall et al., 2003). The Stroop test, a measure of cognitive efficiency and response inhibition, consists of two parts. In Part 1, the participant is asked to read a list of color words as quickly as possible in 1 min. In Part 2, the participant is asked to name the color of the inks that a list of color words is printed with as quickly as possible in 1 min, and the ink and the color word are incongruent. The score ranges from 0 to 112 with higher score indicating better cognitive efficiency and response inhibition (Stroop, 1935). The TMT consists of Parts A and B. Part A is a measure of timed visual sequencing of digits that entails a motor (written) component. Part B, a timed measure of divided attention and set-shifting, introduces alternating attention to digits and letters. At an interval of 1 year between administrations, reliabilities range from .53 to .64 for Part A and .67 to .72 for Part B in older adults (Mitrushina et al., 1994). The COWA is a measure of phonemic fluency that entails cognitive organization and initiation. The participant is asked to name as many words as they can in 1 min beginning with a single letter (*F*, *A*, and *S*). The COWA-FAS has an interrater reliability of .99 (Ross, 2003).

Global cognition was measured using the MMSE (Folstein, Folstein, & McHugh, 1975) and the AD Assessment Scale–Cognitive subscale (ADAS-cog; Rosen, Mohs, & Davis, 1984). The MMSE quantitatively estimates the presence and severity of cognitive impairment with high validity (.68 to .96) and reliability (.80 and .95). MMSE can be administered in 5 to 10 min and assesses seven cognitive domains: orientation to time, orientation to place, registration, attention and calculation, recall, language, and visual construction. The score ranges from 0 to 30 with higher scores indicating better global cognition (Folstein et al., 1975). The ADAS-cog can be administered in 15 min and has 11 items: orientation, word recall, word recognition task, remembering test instructions on word recognition, naming objects and fingers, expressive language, comprehension of spoken language, word finding difficulty, commands, ideational praxis, and construction praxis. The ADAS-cog score ranges from 0 to 70 with higher scores indicating worse global cognition. The ADAS has good interrater (.65-.99) and test–retest reliability (.51-1; Rosen et al., 1984).

QOL was measured by the QOL in AD (QOL-AD; Logsdon, Gibbons, McCurry, & Teri, 2002). The participant is asked to rate 13 items using a 4-point scale: 1 = *poor*, 2 = *fair*, 3 = *good*, or 4 = *excellent*. The items include physical health, energy, mood, living situation, memory, family, marriage, friends, self as a whole, ability to do chores around the house, ability to do things for fun, money, and life as a whole. The score ranges from 13 to 52 with higher scores indicating better QOL. The QOL-AD has a high internal consistency reliability (α = .84) and validity (*r* = .22-.51) in older adults with MMSE > 10 (Logsdon et al., 2002).

Depression was measured using the Geriatric Depression Scale–Short Form (GDS-SF), which contains 15 items and total score of 0 to 15 with higher scores indicating worse depression. The participant is asked to answer “yes” or “no” to each item. The Cronbach's alpha coefficient for GDS-SF was .72 for older adults (Sheikh & Yesavage, 1986).

### Intervention

Details of the cycling intervention have been reported previously (Yu, Savik, Wyman, & Bronas, 2011). All cycling was conducted using Precor™ recumbent stationary cycles (Precor Inc., Woodinville, WA) for 10 to 45 min a session, 3 times a week under the supervision of a certified exercise therapist. One to two participants were supervised at each session.

Cycling was prescribed as individual subjective moderate intensity, which was evidenced by 5 to 6 (tired) on a 10-point modified Borg Rating of Perceived Exertion (RPE) scale, inability to talk, and/or signs and symptoms indicating overexertion. The duration of cycling was 10 min a session initially, which was prolonged by 5 min a session as tolerated until participants could cycle for 45 min at the subjective moderate intensity. To establish the subjective moderate intensity, participants started to cycle at resistance 1 and a low speed (revolutions per minute of 20-40). Resistance and speed were alternatively increased every 2 to 3 min until participants reached a RPE rating of 5 to 6, were unable to talk, and/or showed signs and symptoms indicating overexertion. Heart rate (HR) and the RPE rating were obtained by the exercise therapist every 5 min. The HR at the subjective moderate intensity was documented as the target HR. In addition, participants did 5-min warm-up prior to the 10 to 45 min of cycling and 5-min cool-down after the cycling.

### Procedure

Details of the study procedure have been reported previously (Yu et al., 2011). Briefly, respondents to our recruitment were screened using a three-step procedure: phone screen, in-person interview, and medical verification by primary care provider, for enrollment qualification. Informed consents and assents were obtained from surrogates and participants. Enrolled participants underwent baseline data collection where the undergraduate RA assessed executive function, global cognition, QOL, and depression. Within a week of baseline data collection, participants began their cycling intervention, which was supervised by the exercise therapist. The RA conducted data collection again after participants completed 3- and 6-month cycling, respectively.

### Analysis

All study data were entered into a database via a secure web interface, which was housed on secure servers operated by the university. The database was set up with automatic checks to ensure data entry accuracy and quality. Complete data were abstracted into a Microsoft Excel spreadsheet, which was read into the Statistical Programs for Social Sciences 17.0 for accuracy check using descriptive statistics.

Data were analyzed for participants who completed the study (*n* = 8); there were no missing data for these participants.

Graphs of all measures were generated to get an indication of change from baseline to month 3 and 6. As there were only eight participants, which makes it difficult to determine whether measures are normally distributed, the differences between the three time points were assessed using the Friedman's test. The Friedman's test is a nonparametric, one-way, repeated measures analysis of variance by ranks. If the Friedman's test was significant, post hoc analysis was accomplished using a Wilcoxon Matched-Pairs Signed-Rank Test with a Bonferroni adjustment. In addition, the ES from baseline to month 6 was calculated for each measure using the method of Cohen (Cohen & Erlbaum, 1988), along with a sample size needed to detect a significant difference based on 80% power.

## Results

A total of 61 individuals responded to our recruitment strategies, but only 13 persons had a probable AD diagnosis. Of the 13 people, 2 were further excluded: one could not cycle because of knee pain and one had uncontrolled hypertension. Of the 11 enrolled participants, 3 participants completed 6-month cycling, resulting in a 72.7% retention rate. Reasons for dropout were as follows: nonstudy-related falls and fractures after 3 and 4 weeks of cycling, respectively (*n* = 2), and study-related anxiety attacks after 4 weeks of cycling (*n* = 1). The 3 dropouts were similar to the 8 participants who completed the study in demographic characteristics, but a higher percentage of the dropouts were married (66.7% vs. 12.5%) and lived at home (66.7% vs. 0%). Of the 72 prescribed exercise sessions, participants completed 69 to 72 sessions, indicating an adherence rate of 95.8% to 100%.

The baseline characteristics of the eight participants were as follows: average age of 81.4 years (*SD* = 3.6, range = 77-87); average education of 12.6 years (*SD* = 2.6, range = 8-17); 37.5% men; 62.5% lived in an assisted living facility and 37.5% in group homes; 100% were non-Hispanic White; 50% widowed and 12.5% each for the “married,” “divorced,” “never married,” and “unreported” categories; 87.5% were on AD medications, and 37.5% were on an adrenergic blocking agent.

The scores on three executive function measures show a trend toward improvement from baseline to month 3 and 6 (13.3 vs. 11.6 vs. 11.6 for the EXIT-25, 17.6 vs. 28.0 vs. 20.1 for Stroop Color-Word Test, and 20.6 vs. 26.9 vs. 27.3 for COWA-FAS), but scores for the TMT Part B worsens at month 3 (257.4 s) and 6 (254.1 s) in comparison to baseline (253.4 s). However, none of the above changes from baseline to months 3 and 6 were statistically significant based on the Friedman's tests (see Table 1). ESs for these differences from baseline to 6 months were .48, .27, and .69, respectively. Scores for the TMT Part B appear fairly constant at month 3 (257.4 s) and month 6 (254.1 s) in comparison to baseline (253.4 s, ES baseline to 6 months = .02). Participants are able to reduce the amount of time needed to complete the TMT Part A over time with an ES of .34. A sample size of 70 would be able to detect these differences as significant with 80% power. The projected sample sizes at 80% power for testing the significance of differences in all measures range from 19 to 19,625 (see Table 1).

As Table 1 depicted, there are no statistically significant differences in the two measures of global cognition from baseline to month 3 and 6: MMSE (21.8 vs. 20.4 vs. 19.8, *p* = .67) and ADAS-cog (18.0 vs. 20.1 vs. 22.4, *p* = .20); however, the scores are trending toward worsening global cognition over time. The ESs for the differences in the direction of worsening in the two measures of global cognition from baseline to month 6 were medium to large: MMSE (21.8 vs. 9.8, ES = .54) and ADAS-cog (18.0 vs. 22.4, ES = .70). Based on the ESs, we would be able to detect a significant decline in global cognition with a sample size of 29 for the MMSE and 19 for the ADAS-cog (see Table 1).

The change in QOL is not statistically significant from baseline to month 3 and 6 (see Table 1). The change in QOL resulted in a moderate ES of .31 from baseline to month 6 (see Table 1).A sample size of 84 would have power to detect this difference as significant.

There was a significant linear decrease in depression scores over time (*p* = .026, Freidman's test; see Table 1). Participants reported significantly less depressive symptoms at month 6 compared with baseline (*z* = –1.93, *p* = .05) and month 3 compared with month 6 (*z* = –2.33, *p* = .02); however, their depression levels were low at baseline. The ES for depression is .66 and a sample size of 21 would give a study 80% power to detect a significant change in depression.

## Discussion

A total of 6 months of aerobic exercise intervention has been postulated to be sufficient to generate cognitive benefits in older adults without cognitive impairment (Erickson & Kramer, 2009). A meta-analysis of 30 experimental studies also reported that a mean 23 weeks of exercise for 45 min a session, 3.6 times a week improved global cognition in predominantly nursing homes residents whose cognitive impairment was caused by psychiatric conditions or dementia (Heyn, Abreu, & Ottenbacher, 2004). As a result, we had designed a 6-month, supervised, individualized, moderate intensity of cycling intervention of 10 to 45 min, 3 times a week for community-dwelling older adults with mild to moderate AD in this pilot study. Our pilot study evaluated the changes in executive function, global cognition, QOL, and depression from baseline to month 6 and generated estimates of the ES of various measures over time that could be used to inform a future randomized controlled trial.

Our results revealed some possible changes over time in our participants. First, there appeared to be some indications of improved executive function from baseline to month 3 and 6 as evidenced by better scores on the EXIT-25, TMT Part A, Stroop Color-Word Test, and COWA-FAS with moderate ESs ranging from .27 to .69. The possible improvement is consistent with previous reports that executive function improves the most from aerobic exercise in older adults without cognitive impairment (Colcombe & Kramer, 2003; Smith et al., 2010). Significant improvement in executive function was also demonstrated in older adults with mild cognitive impairment (Baker et al., 2010; van Uffelen et al., 2008).

In contrast, we observed decline in global cognition as measured by the MMSE and ADAS-cog over 6 months. This finding conflicts with the reported gains in global cognition by a meta-analysis in individuals with cognitive impairment and dementias (Heyn et al., 2004) and emerging studies in AD (Kemoun et al., 2010; Palleschi et al., 1996; Rolland et al., 2000). The meta-analysis reported that a mean 23 weeks of exercise intervention for 45 min a session, 3.6 times a week improved global cognition with a moderate ES (Hedge's *g* = .57, *p* ≤ .001) in predominantly nursing home residents with different etiologies for cognitive impairment (Heyn et al., 2004). Studies in AD showed that aerobic exercise of 5 to 12 weeks improved the MMSE scores by 2.3 to 3.5 points with a sample of 35 Europeans (Rolland et al., 2000) and 15 Europeans (Palleschi et al., 1996), respectively. Different samples and doses of aerobic exercise most likely account for the difference in results between our findings and that of other studies.

QOL scores seem to have remained constant over time; however, the scores are fairly high to begin with, making changes difficult to achieve. To our knowledge, this is the first study to have examined the effect of aerobic exercise on QOL in older adults with AD. Finally, our findings indicate a statistically significant improvement in depressive symptoms over time. However, although this is statistical significance, it may not be clinically meaningful because participants had low depressive symptoms even at baseline. This finding is consistent with a previous study, which showed that community-dwelling older adults with AD reduced depression from a caregiver intervention where care-givers learned to implement a comprehensive exercise program and manage dementia behaviors (Teri et al., 2003).

There are several study limitations inherent to a pilot study that needs to be acknowledged. Pilot studies are not powered to detect significant differences. The scope of this pilot study did not allow us to include a control group, which would have resulted in better estimates of change over time. It is also possible that participants may have deteriorated more in their global cognition if our aerobic exercise program was not in place. Furthermore, this study was not designed to test recruitment feasibility, although recruitment was difficult, and we used several recruitment strategies that resulted in only 11 participants enrolled over an 18-month period. To ensure that we had sufficient funds to cover the staff and transportation costs, once participants were enrolled, we limited our recruitment to limited neighborhoods only and did not use recruitment strategies that would have reached larger sample pools. In addition, some participants became frustrated by the cognitive tests or did not appear to make an effort to complete the tasks to their capability, which might have contributed to the patterns we found.

However, this study has several strengths. Our study is the first to measure the impact of aerobic exercise on two new outcome constructs in AD: executive function and QOL. Our exercise therapist and RA were well trained to work with participants with AD. Our exercise intervention was tailored to each individual, started at a low dose, and progressed to the target intensity and duration over time. Our participants reported that the program was enjoyable and kept returning, resulting in a 95.8% to 100% exercise adherence rate. In addition, executive function was comprehensively measured.

Our findings, along with evidence from basic science, imaging studies, and aerobic exercise studies suggest that aerobic exercise is a promising intervention for AD. There are several implications from our study. First, researchers need to better characterize a homogeneous AD sample because wide variations in etiologies for cognitive impairment does jeopardize the comparability and generalizability of results. Second, cognitive domains such as executive function should be comprehensively assessed as the primary outcome. Executive function has been reported to be the most salient to aerobic exercise effects in older adults without AD (Colcombe & Kramer, 2003), and our findings showed some indications of improvement in executive function too. Finally, emerging reports suggest that older adults with AD were significantly less physically active than peers without AD (Burns, Mayo, Anderson, Smith, & Donnelly, 2008; Scarmeas et al., 2009), subjecting them to all the negative health consequences associated with physical inactivity. Even if aerobic exercise does not alter the trajectory of cognitive decline, individuals with AD are likely to achieve many of the same health benefits resulting from aerobic exercise participation as their peers without AD (Hawkins & Wiswell, 2003; Hollenberg, Yang, Haight, & Tager, 2006; Vogel et al., 2009). Based on the magnitude of our ESs, we conclude that aerobic exercise may be able to improve executive function, QOL, and depression in older adults with AD. The results of this study can be used to plan future randomized controlled trials with adequate power to appropriately test these hypotheses.

## Figures and Tables

**Table I:** Comparison of Outcome Measure Scores Over Time (*n* = 8)

Outcome	Baseline	3 Month	6 Month	Friedman's Test *(df 2)*		Effect Size^a^	*n* for 80% Power
					
Variables/Measures	*M* ± *SD*, Range	*M* ± *SD*, Range	*M* ± *SD*, Range	Chi-Square	Significance	Baseline to 6 Months	
Executive function							
EXIT-25	13.3 ± 5.1, 7-21	1 1.6 ± 4.4, 5-18	ll.6 ± 3.1, 8-16	0.75	.69	.48	37
TMT Part A	80.9 ± 32.1, 25-122	64.0 ± 21.8, 34-90	69.3 ± 37.7, 41-137	5.10	.08	.34	70
TMT Part B	253.4 ± 75.9, 112-300	257.4 ± 81.1, 95-300	254.1 ± 66.2, 134-300	0.93	.63	.02	19,625
Stroop Word	109.3 ± 7.4, 91-1 12	107.3 + 13.4, 74-112	107.3 + 13.0, 75-112	0.29	.87	.35	70
Stroop Color-Word	17.6+ 12.3, 3-42	28.0 ± 21.5, 3-75	20.1 ± 9.1, 6-29	3.80	.15	.27	110
COWAT-FAS	20.6 ± 7.7, 9-28	26.9 ± 8.8, 10-38	27.3 ± 12.1, 7-38	2.78	.25	.69	19
F-words	6.6 ± 3.2, 2-10	7.8 ± 2.9, 2-12	9.5 ± 5.4, 0-17	0.84	.66	.58	26
A-words	5.6 ± 3.2, 0-9	9.3 ± 3.8, 4-14	7.5 ± 4.5, 1-14	4.32	.12	.46	37
S-words	8.4 ± 2.3, 5-12	9.9 ± 3.5, 4-15	10.3 + 3.9, 4-15	2.87	.24	.63	22
Global cognition							
MMSE	21.8 ± 5.1, 11-27	20.4 ± 6.6, 6-28	19.8 ± 6.0, 7-27	0.80	.67	.54	29
ADAS-cog	18.0 ± 7.7, 10-32	20.1 ± 9.9, 7-39	22.4 ± 9.3, 7-37	0.33	.20	70	19
QOL							
QOL-AD	42.6 ± 7.2, 31-52	44.6 ± 5.4, 38-52	43.4 ± 6.9, 33-52	0.90	.64	.31	84
Depression							
GDS-SF	2.8 ± 4.3, 0-13	2.0 ± 1.9, 1-6	0.9 ± 1.7, 0-5	7.28	.026*	.66	21

Note: EXIT-25: the Executive lnterview-25, score 0 to 50 with higher score indicating worse executive function; TMT: Trail Making Test, score 0 to 300 with higher score indicating worse executive function; Stroop Word and Color-Word Test: score each 0 to 112 with higher score indicating better executive function; COWA-FAS: Controlled Oral Word Association Test-Letters F, A, S, scores have no range with higher score indicating better executive function; MMSE: Mini-Mental State Examine, score 0 to 30 with higher score indicating better global cognition; ADAS-cog: Alzheimer's Disease Assessment Scale–Cognitive subscale, score 0 to 70 with higher score indicating worse global cognition; QOL-AD: Quality of Life in Alzheimer's Disease, score 13 to 52 with higher score indicating better quality of life; GDS-SF: Geriatric Depression Scale-Short Form, score 0 to 15 with higher score indicating more depression.

a Effect sizes and required sample size for power of 80% were generated using the definition by Cohen.

* The significance level is .05.
